# Skeletal maturity of children with multiple osteochondromas: is diminished stature due to a systemic influence?

**DOI:** 10.1007/s11832-015-0680-x

**Published:** 2015-09-01

**Authors:** Heleen M. Staal, Annemarie L. Goud, Henk-Jan van der Woude, Marianne Adhiambo Witlox, S. John Ham, Simon G. F. Robben, Marjolein H. G. Dremmen, Lodewijk W. van Rhijn

**Affiliations:** Department of Orthopaedic Surgery, Research School Caphri, Maastricht University Medical Centre, P. Debeyelaan 25, P.O. Box 5800, 6202 AZ Maastricht, The Netherlands; Department of Orthopaedic Surgery, Diaconessenhuis, Utrecht, The Netherlands; Department of Radiology, Onze Lieve Vrouwe Gasthuis (OLVG), Amsterdam, The Netherlands; Department of Orthopaedic Surgery, Onze Lieve Vrouwe Gasthuis (OLVG), Amsterdam, The Netherlands; Department of Radiology, Maastricht University Medical Centre, Maastricht, The Netherlands

**Keywords:** Multiple osteochondromas (MO), Multiple hereditary exostoses (MHE), Osteochondroma, Diminished stature

## Abstract

**Background:**

Multiple ostechondromas (MO) is an autosomal dominant inherited disease caused by mutated exostosin genes. It mostly affects the long bones and can lead to growth disturbances, especially disproportionate short stature. Both the local effect on growth plates and the systemic influence of the gene disorder on growth mechanisms might explain the diminished stature.

**Purpose:**

The hypothesis of this study is that the diminished stature in adults with MO is due to a systemic influence, leading to early skeletal maturation and early closure of the growth plate. Therefore, in these patients the skeletal age in adolescence is hypothesized to be higher than the calendar age.

**Methods:**

Radiographs of the left hand were collected from 50 MO-affected children. The skeletal age was calculated using these radiographs according to the Greulich–Pyle bone scale and was compared to the calendar age at the time of radiography.

**Results:**

Children aged 3–12 years had a significantly lower skeletal age compared to their calendar age (*p* = 0.030). Children aged 12–17 years had a significantly higher skeletal age (*p* = 0.019), especially boys. Skeletal maturation in children with MO therefore differs from their peers.

**Conclusion:**

In this study, the skeletal age in younger children with MO is lower than their calendar age. For adolescents, particularly boys, this is reversed, suggesting an earlier or faster closure of the growth plates. These findings support a systemic influence of the gene defect on growth rate.

## Introduction

Multiple ostechondromas (MO), sometimes referred to as multiple hereditary exostoses (MHE) is characterized by the outward growth of cartilage-capped bone tumours called osteochondromas. It is an autosomal dominant inherited disorder caused by mutated exostosin genes (*EXT1* or *EXT2*) [1]. The osteochondromas develop in the first decade of life and cease to grow once the patient reaches skeletal maturity. The long bones are almost always affected but osteochondromas are also found on the scapula, the ribs and the pelvis. MO can lead to growth disturbances including unequal limb length, joint deformity and disproportionate short stature [2–6]. A considerable number of patients suffer from pain or discomfort due to the disorder which significantly affects their quality of life [7]. Diminished or short stature is a common feature in patients with MO. In the majority of patients their height is below normal average height, but within a normal range [7–9]. The origin of this short or diminished stature in MO patients is still unknown. Hypothetically it might be due to a local effect from osteochondromas on the local growth plates or it might be due to a systemic effect.

The local effect of osteochondromas on the growth plate is generally known to lead to leg length discrepancy, Madelungs deformity and other growth disturbances. An analysis by Porter et al. in 2000 showed an inverse correlation between osteochondroma size and relative bone length, suggesting that growth retardation in MO might result from the local effects of enlarging osteochondromas rather than a systemic effect caused by skeletal dysplasia [10]. Several studies have described the systemic influence of the gene defect and its relationship with the growth plate as a possible cause of the short or diminished stature. Jones et al. [32] demonstrated in a mouse genetic model that mice with osteochondromas had shorter femora and tibiae than controls. The volume of the osteochondromas, however, did not correlate with longitudinal shortening. In their model, loss of heterozygosity for *EXT1* was sufficient to drive bone shortening. Other mouse models of MO have provided more information about the pathogenesis of osteochondromas and the role of heparin sulphate (HS) in normal growth plate development [11–13].

Both the local effect of osteochondromas and the systemic HS-influencing effect of the *EXT* genes may explain the diminished stature observed in patients with MO. Furthermore, we also know that MO-affected adults have a shorter stature than expected. However, a study by Clement et al. in 2012 showed that this diminished stature is relative, i.e., in the adolescent age group the stature of MO children seemed to be taller than their peers without the disorder [14]. Hypothetically, the discrepancy between the relatively long stature during adolescence and the diminished stature in adulthood could be due to a systemic influence affecting the maturation of the epiphyses. This in turn could lead to early puberty and early closure of the growth plate. The *EXT* gene disorder affects HS synthesis, which could have a systemic effect or influence on the moment of growth plate closure.

Because skeletal maturation is controlled by hormones, and the same hormones also govern the timing of puberty and might be influenced by systemic gene defects in the *EXT* genes, the hypothesis of this study is that the relatively long stature during adolescence and the diminished stature in adults with MO is due to a systemic influence leading to early maturation of the epiphysis and early closure of the growth plate. If so, the skeletal age in adolescence should be higher than the related calendar age.

The aim of this study is to test the hypothesis that the epiphyses close early in MO-affected children.

## Materials and methods

Data were collected from the joint MO database of the two national tertiary referral centres for MO—the OLVG in Amsterdam and Maastricht University Medical Centre. Fifty patients <20 years of age were selected from this database for whom a radiograph of the left hand had been made for clinical indications between 1995 and March 2015. The Greulich−Pyle method, which has been described previously [15], was used to determine the skeletal age. Briefly, this method uses a standardised set of X-rays of the left hand and wrist, against which the image of the subject is compared.

Two independent radiologists, both highly experienced in skeletal maturity assessment evaluated the X-rays of all 50 patients. Both radiologists were blinded to the original report and the calendar age. When the two independent readings were concordant, i.e., <6 months difference, the average was used. In the case of discordance, the images were re-evaluated by the two radiologists until consensus was reached. The calendar age and skeletal age were recorded in years and months. For statistical analyses, paired *t* test was used.

## Results

There were 23 males and 27 females in the study. Skeletal age could be measured in all patients, despite the presence of osteochondromas in all patients and severe deformation of the wrist in 11 patients. The epiphyseal lines were assessable in all radiographs. The calendar age of the patients varied from 3 years and 9 months to 19 years and 3 months. There were 11 patients aged 3–8 years at the time of radiography, 16 patients aged 8–12 years, 18 patients aged 12–17 years, and five patients aged 17–20 years. The average calendar age was 11 years and 6 months (SD 4.2 years). All results are shown in Table 1.

**Table 1:** Calendar age versus skeletal age (years) of all 50 MO-affected patients

Patient no.	Calendar age	Skeletal age	Patient no.	Calendar age	Skeletal age
1	3.75	3.5	26	11.58	12
2	4.5	3.5	27	11.92	12
3	4.75	3.5	28	12	12
4	5.25	5	29	12	13
5	5.58	5	30	12.67	12
6	5.83	5.83	31	13.5	14.75
7	5.83	5	32	13.75	14
8	5.92	5.75	33	13.83	14.5
9	6.42	6.83	34	14	15
10	6.5	8	35	14.08	14
11	7	6.83	36	14.67	15.5
12	8	6	37	14.75	17
13	8.25	7.83	38	15.33	16
14	8.67	7.83	39	15.33	15.5
15	8.67	8	40	15.42	15.5
16	8.92	7.83	41	15.5	14.75
17	9.67	8.83	42	15.5	15
18	9.75	10	43	15.67	16
19	10	8.83	44	16	16
20	10.08	10	45	17	16
21	10.08	11	46	17.08	17
22	11.08	9	47	18	18
23	11.17	12	48	18.08	18
24	11.42	11.5	49	18.75	19
25	11.58	13	50	19.25	19

**Fig. 1:**
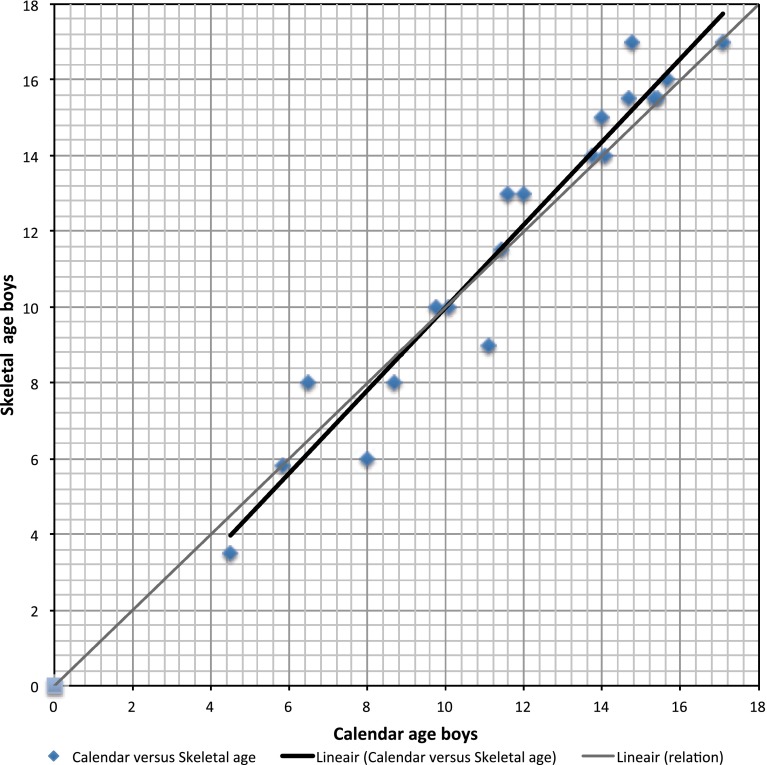
Results of the boys (<17 years) plotted (*blue diamonds*) and their average (*black line*). The *grey line* represents the normal line

**Fig. 2:**
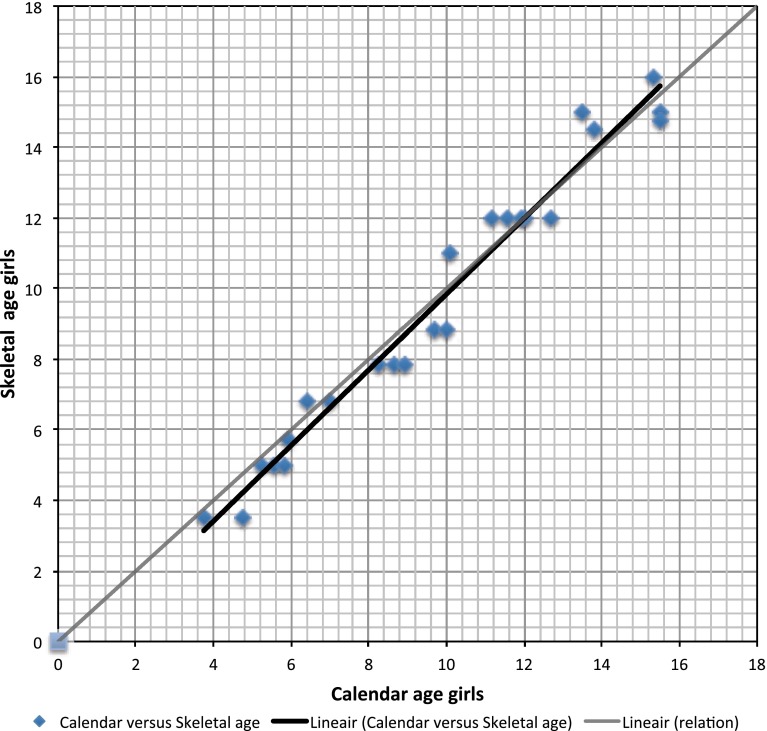
Results of the girls (<17 years) plotted (*blue diamonds*) and their average (*black line*). The grey line represents the normal line

In the MO-affected children <12 years of age the average skeletal age was less than their calendar age in both boys and girls. In this group, the average skeletal age was 7.39 and the average calendar age was 7.79 (*p* = 0.030). In the group aged 12–14 years there seemed to be no difference between the MO-affected children and their unaffected peers. In the children aged >14 years the skeletal age overtook the calendar age; this was more pronounced in boys than in girls (Figs. 1, 2).

Adolescents between 12 and 17 years had a significantly older skeletal age compared to their peers (*p* = 0.019). The average calendar age was 13.95 and the average skeletal age was 14.37. As seen in Fig. 1 for boys and Fig. 2 for girls, the difference was mainly caused by the skeletal age of the boys in this group; the boys between 12 and 17 years had a significantly older skeletal age with a *p* value of 0.011.

There was no statistical difference >17 years of age.

## Discussion

The present study shows that the skeletal age and calendar age in MO children are different from their peers. The skeletal age seems lower in younger children and higher in adolescents. The turning point occurs around the age of 12 at the start of adolescence. Possibly, the growth plates in patients with MO close earlier or faster, particularly in boys.

Our results support the results from a study performed by Clement et al. in 2012 which showed that the final diminished stature of MO patients was relative. They reported that in the adolescent age group the stature of MO children was taller than their peers without the disorder, and >15 years was shorter than their peers, particularly the reduction in leg length [14]. The younger skeletal age in the preadolescent children can explain their relatively longer stature. A younger skeletal age is assessed when the growth plates are wider; the wideness of the growth plates can be caused by the systemic effect of the *EXT* genes.

Several studies have described the systemic influence of the gene defect. Stieber et al. [9] concluded that chondrocytes lacking functional HS influence physeal signalling in general, rather than stealing growth potential focally. Koziel et al. [11] described a genetic mouse line expressing a truncated form of *EXT1* that displayed shortened skeletal elements and fused vertebrae. *EXT* genes belong to a family of glycosyltransferases necessary for the synthesis of the HS. HS regulates signalling of several growth factors. Reduced HS synthesis results in an elevation of Indian hedgehog (Ihh), a protein involved in chondrocyte differentiation. Misexpression of Ihh causes, amongst others, changes in the expression of pro-chondrogenic bone morphogenetic proteins (BMPs) which alter the differentiation of cells in the growth plate as well as the bordering perichondrium [12]. Members of both the BMP and fibroblast growth factor (FGF) families are expressed in growth plate and/or perichondrium and are a part of interactive loops regulating Ihh and parathyroid hormone-related protein expression and the overall growth plate activities [12, 13]. HS is needed to restrain pro-chondrogenic signalling proteins and restricts chondrogenesis. If the HS levels are low this process is disturbed and could lead to increased chondrogenic activity including the activity of cartilage cells in the growth plate, which might lead to wider growth plates. Because HS also influences Ihh activity and Ihh appears to orchestrate chondrocyte proliferation, this might be a second cause for the wider growth plates in preadolescent MO children and thus the relatively young skeletal age [16, 17].

The systemic effect of the genetic disorder can also explain the older skeletal age in adolescent children due to earlier closing of their growth plates. Ihh is essential for normal chondrocyte maturation, regulating both proliferation and differentiation. It also regulates proliferation of chondrocytes by directly controlling the rate of cell division of columnar/proliferative chondrocytes [18, 19]. Inactivation of Ihh in chondrocytes leads to abrupt fusion of the epiphyseal growth plate in mice [20, 21]. In humans an inactivating mutation in Ihh results in acrocapitofemoral dysplasia, which is associated with premature closure of the growth plates [22–26].

Several studies showed that the stature was more severely affected in patients with an *EXT1* mutation [14, 27, 28]. In this study we did not subselect the different gene defect genotypes and therefore cannot contribute to this discussion.

Both mice and human studies showed that the relatively short stature was disproportionate. The sitting height was less affected than leg length indicating more involvement of the limbs than of the axial skeleton [29, 30]. This phenomenon can also be explained by the early closure of the growth plate of the distal femur. The femoral contribution to length is not consistent during growth—approximately 70 % of growth in the femur occurs at the distal growth plate but the proportion of growth occurring in the distal femoral growth plate varies with age, from 55–60 % at approximately 7 years of age to 90 % at 14−16 years [31]. This explains why early closure of the growth plates can lead to relatively short femora and therefore legs, as femoral growth potential is lost.

All of the above seem to support the systemic skeletal dysplasia theory rather than local origin of the disproportional growth. This contradicts the findings of Porter et al. who described a clinical and radiographic analysis of paired bone length in a MO cohort. They found that the local presence of osteochondromas was associated with growth disturbance, and there was an inverse correlation between osteochondroma size and relative bone length. Their conclusion was that growth retardation might result from a local effect [10]. Of note, half of their study patients were adults, and this might have influenced their findings. Their study was performed using radiographs of the forearm to compare the length of the two forearm bones; however, since the epiphysis in these bones does not grow at the same rate, the growth might be influenced at a different pace. Moreover, the position of the osteochondromas might play an important role in the forearm, since the interosseous membrane connects the two bones and the relative position of the osteochondroma in relation to this membrane might play a role in growth disturbance. The forearm bones cannot be measured as two separate bones because of their close relationship. Furthermore, the finding of the inverse correlation could be due the severity of the disease rather than the actual size of osteochondromas. More and bigger osteochondromas means more severe MO and thus more influence of the gene dysfunction on the biochemical setup. A mouse study by Jones also does not support the relationship between the volume of osteochondromas and the growth plate influence [32].

An obvious limitation to the present study is the inter-individual variation in the skeletal age. Different population groups mature at different speeds. For instance, healthy living children mature faster than children living under poor conditions. Furthermore, the standard deviation of skeletal age at a given age can vary by approximately 1 year. The number of patients in this study is relatively low to compensate for this large deviation. For future studies we advocate selecting larger numbers of patients and dividing them by their genotype. In this way, the underlying gene defect and its relationship on growth disturbance can be analysed.

This study has shown the skeletal age in MO patients differs from their calendar age. This is of direct clinical relevance in the planning of epiphysiodesis in leg length discrepancy and hemi-epihysiodesis in axial deformities in MO patients, especially boys. Individual longitudinal follow-up of bone growth is advised.

## Conclusion

The skeletal age in younger children with MO is lower than their calendar age, while for adolescent boys it is higher. The turning point occurs around the start of adolescence. This phenomenon may explain the diminished stature in MO patients. These findings support a systemic influence of the gene defect on growth rate.
